# Tendência Temporal das Internações Hospitalares por Insuficiência Cardíaca no Brasil

**DOI:** 10.36660/abc.20240505

**Published:** 2025-05-19

**Authors:** José Marcos Girardi, Isadora Araújo Girardi, Ana Clara Silva Nascimento, Daniel Monteiro de Lauro Silva, Luisa Venture Gibaile Soares, Sarah Alessandrini Lauriano Dias, Sarah Quick Lourenço de Lima, Flávia Araújo Girardi

**Affiliations:** 1 Sociedade Brasileira de Cardiologia Juiz de Fora MG Brasil Sociedade Brasileira de Cardiologia – Cardiologia, Juiz de Fora, MG – Brasil; 2 Faculdade de Ciências Médicas e da Saúde de Juiz de Fora Juiz de Fora MG Brasil Faculdade de Ciências Médicas e da Saúde de Juiz de Fora, Juiz de Fora, MG – Brasil; 3 Universidad Maimónides Facultad de Medicina Buenos Aires Argentina Universidad Maimónides - Facultad de Medicina, Buenos Aires – Argentina; 4 Universidade Federal de Juiz de Fora Juiz de Fora MG Brasil Universidade Federal de Juiz de Fora, Juiz de Fora, MG – Brasil

**Keywords:** Insuficiência Cardíaca, Hospitalização, Mortalidade

## Abstract

**Fundamento:**

A insuficiência cardíaca é uma pandemia global e causa de redução significativa da qualidade de vida, com impacto nos gastos hospitalares, sendo importante conhecer a tendência temporal das internações e mortalidade para traçar estratégias de enfrentamento.

**Objetivo:**

O objetivo deste estudo foi descrever a tendência temporal das internações hospitalares por insuficiência cardíaca e mortalidade durante as internações entre 2000 e 2021 no Brasil.

**Métodos:**

Foi realizado estudo de tendência temporal das taxas de internação e mortalidade durante internações, utilizando-se dados do Departamento de Informática do Sistema Único de Saúde, por meio de regressão segmentada joinpoint. Foram calculadas as variações percentuais anuais das taxas com os respectivos intervalos de confiança de 95% e nível alfa de significância de 0,05.

**Resultados:**

Foram analisadas 2.851.437 internações em homens e 2.749.424 em mulheres entre 2000 e 2021 no Brasil. Observou-se redução percentual anual das taxas de internação em homens de 6,7% a 8,1% e, em mulheres, redução de 7,5% a 8,3%. Observou-se aumento percentual anual das taxas de mortalidade em homens de 1,8% a 3,6% e, em mulheres, aumento de 3,1% a 3,5%.

**Conclusão:**

Observou-se redução das taxas de internação por insuficiência cardíaca e aumento das taxas de mortalidade em todas as faixas etárias avaliadas para ambos os sexos entre 2000 e 2021 no Brasil. Estes resultados podem refletir melhor controle ambulatorial da doença e internação apenas para casos mais graves, mas ressaltamos a necessidade de abordagem continuada dos fatores de risco para a doença.

## Graphical abstract

**Figura Central:**
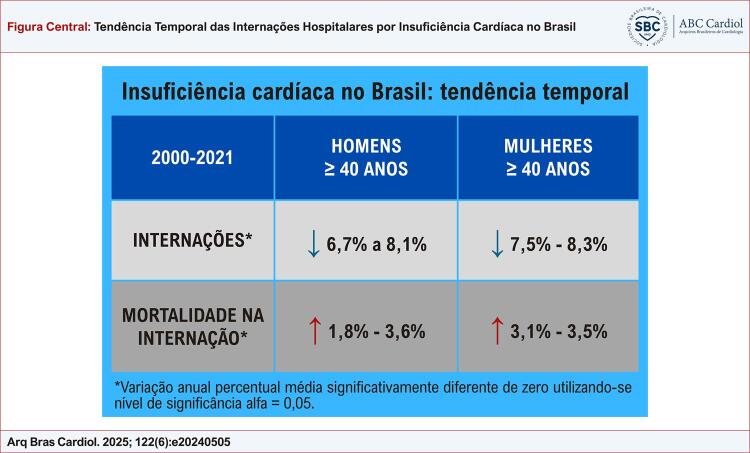
Tendência Temporal das Internações Hospitalares por Insuficiência Cardíaca no Brasil

## Introdução

A insuficiência cardíaca (IC) é definida como uma síndrome clínica na qual o coração é incapaz de bombear o sangue para atender às necessidades metabólicas tissulares ou é capaz de fazê-lo apenas com elevadas pressões de enchimento.^1^ As causas mais comuns de IC incluem cardiopatia isquêmica, infarto do miocárdio, hipertensão arterial sistêmica e valvopatias.^2^

A IC é considerada uma pandemia global, tendo acometido em torno de 64 milhões de pessoas no mundo em 2017^3^ e é causa de redução significativa da qualidade de vida, com impacto importante nos gastos hospitalares e atendimentos de urgência.^1^ Nos Estados Unidos da América (EUA), embora o número absoluto de pacientes com IC tenha crescido, parcialmente como resultado do aumento do número de idosos, a incidência da IC diminuiu. Houve redução das hospitalizações por IC nos EUA até 2012. Porém, observou-se aumento entre 2013 e 2017, tendo sido registradas 1,2 milhão de hospitalizações por IC entre 924.000 pacientes com diagnóstico da doença em 2017, representando um aumento de 26,0% no período.^2^

Nos EUA, aproximadamente 115 milhões de pessoas têm hipertensão arterial, 100 milhões têm obesidade, 26 milhões têm diabetes e 125 milhões têm doença cardiovascular (DCV) aterosclerótica. Esses são fatores bastante conhecidos e de alto risco populacional atribuível para o desenvolvimento de IC. Portanto, uma grande proporção da população pode ser categorizada como estando em risco para IC.^2^ Na América Latina, um perfil clínico distinto é encontrado, com potenciais fatores de risco resultantes de um baixo investimento na saúde, inadequado acesso ao atendimento e acompanhamento insuficiente nos serviços em nível primário ou terciário.^1^

Neste contexto, no Brasil, dados anuais compilados e pesquisas sobre epidemiologia das DCV mostram que estas costumam ser a principal causa de morte, sendo a doença de artéria coronária a causa número um, seguida por acidente vascular cerebral. Porém, em 2021, a COVID-19 tornou-se a principal causa em ambos os sexos. Esses dados epidemiológicos, apontados pela Estatística Cardiovascular – Brasil, revelam que diagnósticos autorreferidos de hipertensão arterial, de hipercolesterolemia e de diabetes mellitus ocorreram em adultos na ordem de 26,3% (2021), 14,6% (2019), e 9,1% (2021), respectivamente. Houve redução no número de casos de diabetes desconhecidos no Brasil, o que pode ter ocorrido devido à maior taxa de rastreamento e maior acesso ao diagnóstico em alguns segmentos da população. A obesidade em 2021 foi registrada em 22,4%, com tendência de aumento de 2006 a 2021. Já a prevalência de tabagismo nesse segmento populacional apresentou redução de 0,7% entre 2019 e 2021 em ambos os sexos. Com relação à atividade física, observa-se que, apesar do conhecimento crescente em relação aos benefícios cardiovasculares de uma prática regular e uma tendência à redução da inatividade física entre os brasileiros nos últimos anos, quase metade da população não alcança o nível mínimo recomendado de atividade física.^4^

A despeito de avanços na terapêutica da IC, a síndrome mantém-se como uma patologia grave, cuja sobrevida após 5 anos de diagnóstico pode chegar a apenas 35,0%.^1^ No Brasil, a má aderência à terapêutica básica para IC é a principal causa de rehospitalizações e da elevada taxa de mortalidade intra-hospitalar.^5^

O objetivo deste estudo foi descrever a tendência temporal das internações hospitalares por IC, bem como da mortalidade durante a internação, entre 2000 e 2021 no Brasil, analisando sua distribuição por faixas etárias.

## Métodos

### Tipo de estudo e fontes dos dados

Trata-se de estudo ecológico de tendência temporal das taxas de internação por IC e taxas de mortalidade durante a internação entre 2000 e 2021 no Brasil.

Foram utilizados os dados do Departamento de Informática do Sistema Único de Saúde (DATASUS), o qual auxilia diretamente o Ministério da Saúde com provimento de suporte aos sistemas de informação.^6^

### População de estudo e variáveis de interesse

A população do estudo abrangeu as internações hospitalares em ambos os sexos por IC entre 2000 e 2021 no Brasil. O período foi escolhido com base na disponibilidade de dados de base populacional no DATASUS. Foram selecionadas as faixas etárias a partir de 40 anos de idade, tendo em vista que representaram mais de 95,0% das internações por IC em adultos.

Foram utilizados os dados de morbidade hospitalar geral do Sistema Único de Saúde (SUS), por local de residência, extraídos da ferramenta de consulta TABNET do DATASUS.^7^ Foram selecionadas as variáveis sexo, faixas etárias (agregadas em períodos de 10 anos) a partir de 40 anos de idade, número de internações e taxas de mortalidade durante a internação para cada ano separadamente. Em lista de morbidades CID-10,^8^ foi selecionado o termo “insuficiência cardíaca”. Os dados de população residente foram extraídos da seção de variáveis demográficas e econômicas da ferramenta TABNET, estratificados por sexo, faixas etárias e anos.

A variável de desfecho taxa de mortalidade na internação foi extraída diretamente da ferramenta TABNET, já calculada para cada 100 internações. Trata-se da razão entre a quantidade de óbitos e o número de autorizações de internação hospitalar (AIH) aprovadas, computadas como internações, no período, multiplicada por 100.

A variável de desfecho taxa de internação foi calculada da seguinte forma:


$$\frac{\text{ Número de internações por IC por faixa etária, sexo e ano }\;\times 10.000}{\text{ Número de pessoas residentes da mesma faixa etária, sexo e ano }}$$

Para fins de correção do número de internações por causas mal definidas (CMD), foi realizado o ajuste proposto por Mathers et al. *,* ^9^ com base no qual foi calculado o percentual de internações por causas mal definidas (PICMD):


$$\frac{Total\;de\;internações\;–\;internações\;por\;causas\;externas}{(Total\;de\;internações\;–\;internações\;por\;causas\;externas)\;–\;internações\;por\;CMD}$$

Após, foi calculado um fator de correção (FC) por faixa etária e ano, o qual foi multiplicado pelo número de internações corrigidas:

FC das internações por CMD:


$$FC=1+\frac{\left(PICMD-1\right)}{2}$$

As referidas correções foram realizadas para cada sexo, faixa etária e ano. As causas mal definidas são apresentadas na CID-10 no Capítulo XVIII, entre os códigos R00 e R99. Já as internações por causas externas são apresentadas diretamente na seção de morbidade hospitalar na ferramenta TABNET.

### Análises de dados

A variação percentual anual (APC, do inglês *annual percentage change* ) das taxas de internação por IC e das taxas de mortalidade na internação foram calculadas através de modelagem pelo método de regressão *joinpoint* , utilizando o ano calendário como variável independente, segundo faixas etárias. Tal método permite identificar os pontos de mudança de tendência no tempo e, a partir da definição do melhor modelo, são calculadas as APC para cada segmento. Dessa forma, pode-se descrever e quantificar a tendência, avaliando se a mesma é estatisticamente significativa.

Como medida que sintetiza a tendência em todo o período, foi utilizada a variação percentual anual média (AAPC, do inglês *average annual percentage change* ). Para APC e AAPC foram estimados intervalos de confiança de 95% e utilizado nível de significância alfa igual a 0,05. Foram selecionadas as opções de homoscedasticidade, transformação logarítmica da variável dependente e modelo de ajustamento de erros correlacionados baseado nos dados. Para as análises de tendência, foram utilizados períodos de um ano e os resultados foram apresentados sob a forma de tabelas e gráficos de regressão segmentada.

Foram utilizados os programas Excel (Microsoft Office Home and Student 2019) para entrada dos dados e Joinpoint Regression Software versão 4.9.0.0^10^ para a análise de regressão segmentada.

## Resultados

Foram registradas 184.715 internações por IC entre homens e 187.108 entre mulheres no ano de 2000 no Brasil, sendo que faixa etária de 60 a 79 anos representou, respectivamente, 55,4% e 53,9% dos números absolutos de internações. Entretanto, as taxas de internação foram maiores em indivíduos com 80 anos ou mais, 479,1 em homens e 421,9 em mulheres. As taxas de mortalidade na internação também foram maiores nessa faixa etária, 9,2 entre homens e 10,3 entre mulheres. Já em 2021, foram registradas 81.744 internações por IC entre homens e 75.016 entre mulheres. A faixa etária de 60 a 79 anos representou 54,7% das internações em homens e a faixa etária de 70 anos ou mais representou 57,0% das internações em mulheres. Os indivíduos de 80 anos ou mais apresentaram as maiores taxas de internação, 88,7 em homens e 75,1 em mulheres, e de mortalidade, 20,0 em homens e 20,3 em mulheres (Tabela Suplementar).

As taxas de internação por IC apresentaram redução estatisticamente significativa em todas as faixas etárias avaliadas em ambos os sexos entre 2000 e 2021. As médias de redução percentual anual das taxas de internação por IC em homens variaram de 6,7% para a faixa etária de 40 a 49 anos a 8,1% para a faixa de 80 anos ou mais. Entre as mulheres, as referidas médias apresentaram redução de 7,5 entre 70 e 79 anos a 8,3% entre 50 e 59 anos ( Figura Central e Tabela 1 ).

**Figura 1:**
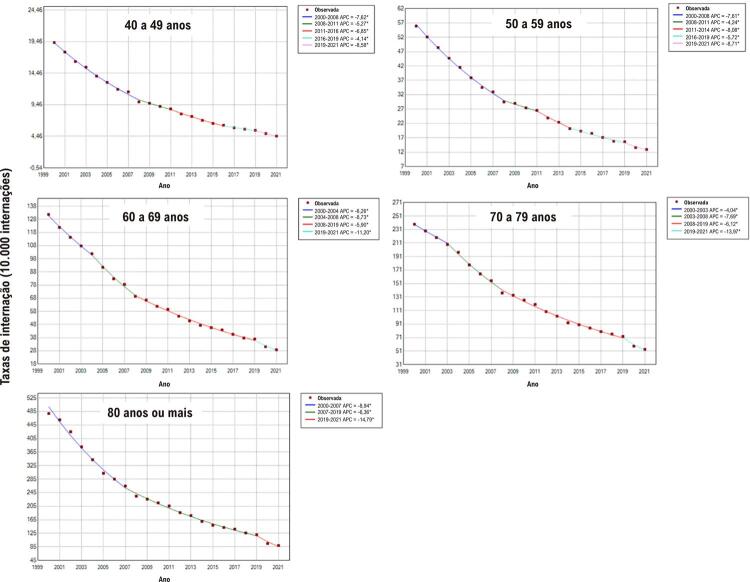
– Valores observados e estimados por modelo de regressão joinpoint das taxas de internação por insuficiência cardíaca em homens, por faixas etárias, entre 2000 e 2021 no Brasil. Pontos: valores observados; linhas contínuas: valores estimados. Valores de APC (variação percentual anual) e respectivos intervalos de confiança 95%: 40 a 49 anos: –7,6%* (–8,0; –7,3); –5,3%* (–7,1; –4,7); –6,8%* (–8,0; –6,4); –4,1%* (–5,0; –3,4); –8,6%* (–10,0; –6,8). 50 a 59 anos: –7,6%* (–7,9; –7,4); –4,2%* (–5,5; –3,7); –8,1%* (–8,7; –7,0); –5,7%* (–6,1; –4,5); –8,7%* (–10,4; –6,9). 60 a 69 anos: –6,3%* (–6,9; –4,7); –8,7%* (–9,8; –7,9); –5,9%* (–6,1; –5,6); –11,2%* (–12,7; –8,9). 70 a 79 anos: –4,0%* (–5,5; –0,8); –7,7%* (–9,2; –6,9); –6,1%* (–6,4; –5,3); –14,0%* (–16,1; –10,7). 80 anos ou mais: –8,9%* (–10,0; –8,2); –6,4%* (–6,7; –5,8); –14,8%* (–17,3; –10,9). *APC significativamente diferente de zero utilizando-se nível de significância alfa = 0,05. Elaborado pelos autores (2024).

As taxas de internação por IC apresentaram redução variada conforme o período e a faixa etária. No primeiro decênio avaliado, as reduções anuais mais expressivas foram observadas nas faixas etárias de 80 anos ou mais (8,9% entre 2000 e 2007) e 60 a 69 anos (8,7% entre 2004 e 2008) para a população masculina. Já entre as mulheres, foram observadas reduções mais expressivas nas faixas etárias de 40 a 49 anos (10,6% entre 2000 e 2006), 50 a 59 anos (9,9% entre 2000 e 2007), 60 a 69 anos (9,0% entre 2000 e 2008) e 80 anos ou mais (10,5% entre 2000 e 2005). No segundo decênio, em geral, as reduções das taxas de internação por IC foram menores, exceto para homens de 50 a 59 anos (8,1% entre 2011 e 2014). Entretanto, as reduções mais acentuadas das taxas de internação foram observadas entre 2019 e 2021, superiores a 11,0% ao ano a partir de 60 anos de idade para os homens e 12,0% a partir de 50 anos para as mulheres ( Figuras 1 e 2 ).

**Figura 2:**
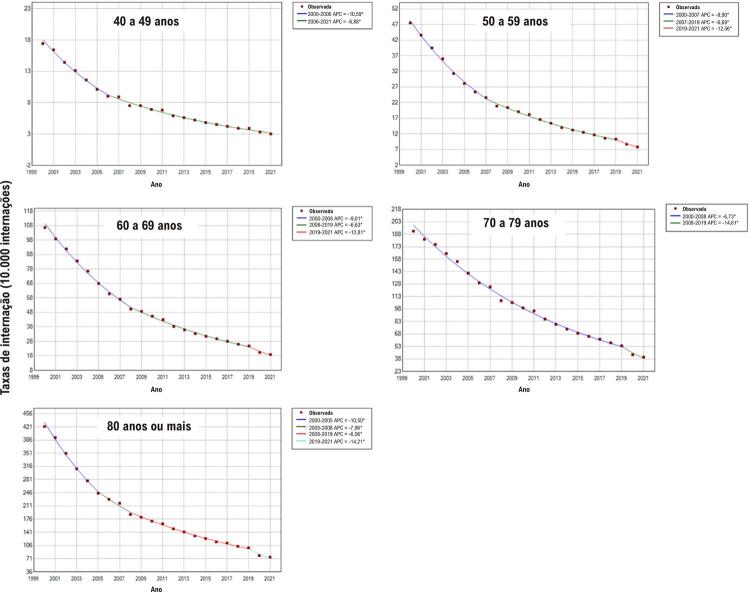
– Valores observados e estimados por modelo de regressão joinpoint das taxas de internação por insuficiência cardíaca em mulheres, por faixas etárias, entre 2000 e 2021 no Brasil. Pontos: valores observados; linhas contínuas: valores estimados. Valores de APC (variação percentual anual) e respectivos intervalos de confiança 95%: 40 a 49 anos: –10,6%* (–12,0; –9,6); –6,9%* (–7,1; –6,6). 50 a 59 anos: –9,9%* (–10,8; –9,2); –6,7%* (–6,9; –6,2); –12,6%* (–15,0; –9,3). 60 a 69 anos: –9,0%* (–9,9; –8,4); –6,6%* (–6,9; –5,8); –12,8%* (–15,6; –9,0). 70 a 79 anos: –6,7%* (–6,9; –6,5); –14,6%* (–17,4; –10,2). 80 anos ou mais: –10,5%* (–12,6; –8,7); –8,0%* (–11,0; –5,1); –6,1%* (–6,4; –5,0); –14,2%* (–16,7; –10,9). *APC significativamente diferente de zero utilizando-se nível de significância alfa = 0,05. Elaborado pelos autores (2024).

As taxas de mortalidade durante a internação por IC apresentaram elevação estatisticamente significativa em todas as faixas etárias avaliadas em ambos os sexos entre 2000 e 2021. As médias de aumento percentual anual em homens variaram de 1,8% para a faixa etária de 40 a 49 anos a 3,6% para a faixa de 80 anos ou mais. Entre as mulheres, as referidas médias variaram de 3,1% para a faixa de 80 anos ou mais a 3,5% entre 60 e 79 anos ( Figura Central e Tabela 1 ).

**Tabela 1:** – Variação percentual anual média das taxas de internação e de mortalidade na internação por insuficiência cardíaca em homens e mulheres, por faixas etárias, entre 2000 e 2021 no Brasil

Faixa etária/taxas	Homens			Mulheres		
	AAPC	(IC95%)	Tendência	AAPC	(IC95%)	Tendência
40 a 49 anos						
Internação	–6,7%*	(–6,8; –6,6)	Decrescente	–8,0%*	(–8,2; –7,7)	Decrescente
Mortalidade	1,8%*	(1,5; 2,1)	Crescente	3,4%*	(3,1; 3,8)	Crescente
50 a 59 anos						
Internação	–6,9%*	(–7,0; –6,8)	Decrescente	–8,3%*	(–8,6; –8,1)	Decrescente
Mortalidade	2,7%*	(2,3; 2,8)	Crescente	3,4%*	(3,1; 3,7)	Crescente
60 a 69 anos						
Internação	–7,0%*	(–7,2; –6,9)	Decrescente	–8,1%*	(–8,4; –7,9)	Decrescente
Mortalidade	3,3%*	(2,9; 3,5)	Crescente	3,5%*	(3,0; 3,7)	Crescente
70 a 79 anos						
Internação	–7,0%*	(–7,2; –6,7)	Decrescente	–7,5%*	(–7,8; –7,3)	Decrescente
Mortalidade	3,4%*	(3,1; 3,5)	Crescente	3,5%*	(3,0; 3,7)	Crescente
80 anos ou mais						
Internação	–8,1%*	(–8,3; –7,8)	Decrescente	–8,2%*	(–8,4; –8,0)	Decrescente
Mortalidade	3,6%*	(3,4; 3,9)	Crescente	3,1%*	(2,8; 3,3)	Crescente

Internação: taxas de internação por 10.000 habitantes; mortalidade: taxas de mortalidade durante a internação por 100 internações. AAPC: variação percentual anual média (2000 a 2021); IC95%: intervalo de confiança 95%. *AAPC significativamente diferente de zero utilizando-se nível de significância alfa = 0,05. Elaborado pelos autores (2024).

**Figura 3:**
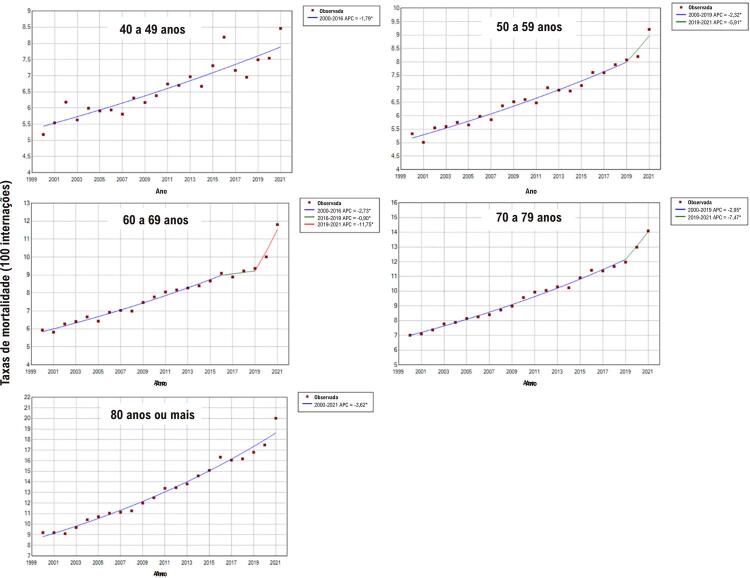
– Valores observados e estimados por modelo de regressão joinpoint das taxas de mortalidade durante as internações por insuficiência cardíaca em homens, por faixas etárias, entre 2000 e 2021 no Brasil. Pontos: valores observados; linhas contínuas: valores estimados. Valores de APC (variação percentual anual) e respectivos intervalos de confiança 95%: 40 a 49 anos: 1,8%* (1,5; 2,1). 50 a 59 anos: 2,3%* (1,2; 4,3); 5,9%* (2,3; 8,1). 60 a 69 anos: 2,7%* (2,4; 6,1); 0,9% (–0,9; 2,7); 11,8%* (6,2; 15,3). 70 a 79 anos: 2,9%* (2,6; 3,1); 7,5%* (3,5; 9,2). 80 anos ou mais: 3,6%* (3,4; 3,9). *APC significativamente diferente de zero utilizando-se nível de significância alfa = 0,05. Elaborado pelos autores (2024).

Para a população masculina, observou-se aumento anual mais pronunciado das taxas de mortalidade na internação nas faixas etárias de 60 a 69 anos (11,8%) e 70 a 79 anos (7,5%) entre 2019 e 2021. Entre as mulheres, o aumento anual mais expressivo foi registrado nas faixas etárias de 50 a 59 anos (7,6%), 60 a 69 anos (8,9%) e 70 a 79 anos (9,3%) no mesmo período ( Figuras 3 e 4 ).

**Figura 4:**
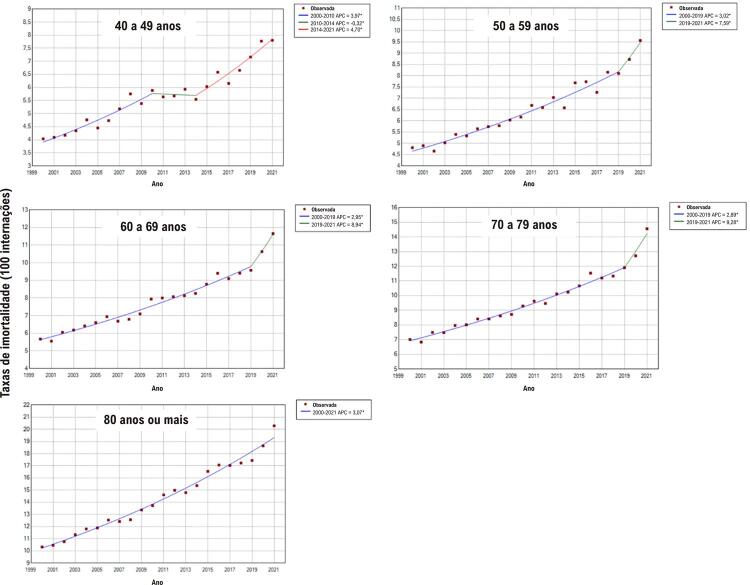
– Valores observados e estimados por modelo de regressão joinpoint das taxas de mortalidade durante as internações por insuficiência cardíaca em mulheres, por faixas etárias, entre 2000 e 2021 no Brasil. Pontos: valores observados; linhas contínuas: valores estimados. Valores de APC (variação percentual anual) e respectivos intervalos de confiança 95%: 40 a 49 anos: 4,0%* (3,4; 5,3); –0,3% (–3,1; 2,1); 4,7%* (3,5; 8,4). 50 a 59 anos: 3,0%* (2,4; 3,2); 7,6%* (3,2; 10,3). 60 a 69 anos: 2,9%* (2,3; 3,2); 8,9%* (3,3; 11,7). 70 a 79 anos: 2,9%* (2,5; 3,1); 9,3%* (3,5; 11,4). 80 anos ou mais: 3,1%* (2,8; 3,3). *APC significativamente diferente de zero utilizando-se nível de significância alfa = 0,05. Elaborado pelos autores (2024).

## Discussão

Nosso estudo constatou, entre 2000 e 2021, uma tendência decrescente das taxas de internação por IC em todas as faixas etárias avaliadas tanto para homens (6,7% a 8,1%) quanto para mulheres (7,5% a 8,3%). Já as taxas de mortalidade durante a internação mostraram tendência crescente no mesmo período em todas as faixas etárias avaliadas tanto para homens (1,8% a 3,6%) quanto para mulheres (3,1% a 3,5%).

A IC é um fardo econômico e de saúde pública crescente para todo o mundo, principalmente devido ao envelhecimento populacional. A IC e o acidente vascular encefálico foram as causas mais frequentes de internações hospitalares por doenças do aparelho circulatório em ambos os sexos em 2021 no Brasil, cada uma representando aproximadamente 17,0% do total. Dentre as internações por todas as causas, foram atribuídos à IC 2,6% dos casos no mesmo ano.^7^

De acordo com o DATASUS, foram registradas 3.454.570 hospitalizações por IC de 2008 a 2021, mais de um terço do total de admissões clínicas relacionadas a condições cardiovasculares. Entretanto, houve redução no número de admissões clínicas por IC, que passou de 298.474 (157 por 100.000) em 2008 para 181.441 (85 por 100.000) em 2021, sendo tal redução uniforme ao longo dos anos, o que não se correlacionou com redução de custos assistenciais. O número reduzido de admissões e as despesas aumentadas representaram maiores custos por admissão no período observado (de R$ 912 em 2008 para R$ 1.787 em 2021).^4^

Os fatores precipitantes mais comuns para a hospitalização de pacientes com descompensação aguda da IC compreendem a síndrome coronária aguda, fibrilação atrial e outras arritmias, doença cardíaca superposta (como endocardite), infecções agudas (pneumonia, infecção do trato urinário), não aderência à dieta ou medicação, anemia, hiper ou hipotireoidismo, medicamentos com aumento da retenção sódica (como os anti-inflamatórios não esteroidais) e medicamentos com efeito inotrópico negativo (por exemplo, o verapamil).^2^

Observamos em nossos resultados uma redução das taxas de internação por IC, o que poderia ser interpretado como resultado de políticas de saúde bem sucedidas no país. Ressalta-se, entretanto, que a presença dos fatores de risco clássicos (hipertensão arterial sistêmica, dislipidemia, obesidade, sedentarismo, tabagismo, diabetes mellitus e histórico familiar de DCV) aumenta a probabilidade de DCV, notadamente de etiologia isquêmica, enfatizando a importância da prevenção primária e secundária. Em estudo brasileiro realizado entre 2011 e 2012, a etiologia isquêmica da IC predominou nos pacientes provenientes das regiões Sul (33,6%), Sudeste (32,6%) e Nordeste (31,9%). Na região Norte, predominou a etiologia hipertensiva (37,2%) e, no Centro-Oeste, a doença de Chagas (42,4%).^5^ A doença cardíaca isquêmica mantém-se como a principal causa de óbitos em ambos os sexos no Brasil, apesar do declínio da prevalência e incidência da doença nos últimos 20 anos.^11^ As maiores taxas de mortalidade por doença cardíaca isquêmica foram atribuíveis à hipertensão arterial sistêmica em 2021 no Brasil, em torno de 37,6 por 100.000 habitantes, seguido das taxas atribuíveis a níveis séricos elevados de LDL ( *low-density lipoprotein* ) colesterol, com 26,7 por 100.000 habitantes. Entretanto, as referidas taxas mantiveram-se estáveis entre 1990 e 2021. Já as taxas de mortalidade por doença cardíaca hipertensiva tiveram como principais fatores de risco associados a hipertensão arterial sistêmica (13,3 por 100.000 habitantes) e o índice de massa corporal elevado (7,5 por 100.000 habitantes), porém, com aumento da mortalidade de, respectivamente, 17,0% e 36,0% entre 1990 e 2021.^12^ Em relação à doença de Chagas, fatores socioeconômicos e ambientais, condições de habitação e saneamento ainda são elementos que impactam na transmissão da doença no Brasil.^13^ Assim, embora alguns fatores de risco mostrem-se estabilizados, outros apresentaram elevação com impacto na mortalidade por doenças cardíacas que levam à síndrome da IC.

Outro aspecto importante a ser considerado é o advento de fármacos capazes de modificar o curso clínico da síndrome, reduzindo o risco de internação. O tratamento da IC até 1987 incluía dieta hipossódica, diuréticos para as manifestações de congestão, digoxina e repouso. Entretanto, nenhuma destas recomendações possuía fundamentação epidemiológica-clínica, mas eram apenas baseadas no conhecimento fisiopatológico à época, no efeito farmacológico das medicações e no bom-senso.^14^ Em 1987, iniciou-se uma nova era no tratamento da IC, com a publicação do primeiro ensaio clínico randomizado utilizando o enalapril. Esta nova droga teria a capacidade de inibir o sistema renina-angiotensina-aldosterona, um eixo hormonal envolvido na causa, manutenção e incremento de risco da IC.^15^ Em 1996, foram identificados os betabloqueadores como a segunda classe de drogas capazes de mudar a história natural da doença.^16^ Em 1999, a espironolactona em baixas doses, explorando mais seu efeito hormonal do que o efeito diurético, foi a próxima droga a mostrar benefício definitivamente comprovado sobre o prognóstico da IC.^17^ Seguiram-se os inibidores da neprilisina e receptores da angiotensina em 2014^18^ e os inibidores do co-transportador sódio-glicose 2 em 2019,^19 , 20^ considerados fármacos inovadores no tratamento da IC por terem demonstrado importantes reduções no risco de hospitalizações em torno de 40,0% a 45,0% e 30,0%, respectivamente.^14^ Atualmente, estão disponíveis no SUS diversas medicações para tratamento da IC, inclusive aqueles com impacto na sobrevida, como inibidores da enzima conversora de angiotensina, betabloqueadores e antagonistas da aldosterona.^21^

No presente estudo, também foram observadas reduções mais expressivas das taxas de internação por IC entre 2019 e 2021, fato que pode ter sido decorrente da pandemia por SARS-CoV-2. Medidas visando reduzir a transmissão da COVID-19 afetaram a organização dos serviços de saúde, com redução dos atendimentos presenciais e recomendação para que a população procurasse o serviço somente em casos de extrema necessidade.^22 , 23^

Constatamos em nosso estudo que houve aumento das taxas de mortalidade na internação por IC entre 2000 e 2021 no país, o que poderia refletir o fato de que apenas casos mais graves foram internados ou a alta frequência de comorbidades, principalmente em indivíduos idosos.^1^ No Brasil, observou-se taxas de óbito intra-hospitalar por IC duas vezes maiores em relação a registros americanos e europeus.^4^ As mortes atribuíveis a doenças cardíacas têm aumentado globalmente devido, em parte, ao aumento do reconhecimento, diagnóstico e documentação de cardiomiopatias específicas e de casos de cardiotoxicidade.

Observamos também elevação mais expressiva das taxas de mortalidade na internação por IC na faixa etária de 60 a 79 anos entre 2019 e 2021. Em 2020, verificou-se redução do número absoluto de óbitos por DCV no Brasil, porém com aumento da mortalidade hospitalar em todas as regiões do país, a qual pode estar relacionada a diversos fatores. Mudanças no sistema de saúde durante a pandemia, como redirecionamento de equipes para atender pacientes com COVID-19, implicaram em redução de consultas e cirurgias eletivas. Ademais, a demora na busca por atendimento médico e os efeitos prejudiciais da infecção por SARS-CoV-2 no sistema cardiovascular podem ter contribuído para o aumento da descompensação clínica de pacientes cardiopatas.^22^

Com a pandemia, pesquisadores estão obtendo melhores informações sobre a infecção e a lesão miocárdica relacionada à inflamação e miocardite. Com o aumento da capacidade de detectar lesão miocárdica por métodos diagnósticos bioquímicos e de imagem, além de uma crescente conscientização sobre os padrões de cardiotoxicidade e injúria, incluindo inflamação, a incidência da IC provavelmente continuará a aumentar.^2^

No contexto do controle dos fatores de risco, diagnóstico precoce, aderência ao tratamento da IC e a atenção primária à saúde do SUS desempenham um papel fundamental em virtude de seu caráter longitudinal do cuidado.^15^ Também, a implementação de políticas de saúde, como o estímulo aos hábitos de vida saudáveis, medidas de prevenção primária e secundária e o acesso ao tratamento de eventos cardiovasculares, constitui fator essencial para o controle das DCV em todo o mundo.

Dentre as limitações deste estudo, destaca-se que a caracterização da IC na base de dados do DATASUS pode estar subestimada, uma vez que a internação pode ter sido decorrente da doença de base que levou à IC ou outra comorbidade e, portanto, ser registrada com código CID-10 diverso. Além disto, atenta-se para a possibilidade de subnotificação, pois o diagnóstico de IC pode não identificar adequadamente todos os casos, uma vez que as análises de imagem e bioquímicas não estão disponíveis em todos os serviços e regiões do país.

Apesar das limitações inerentes ao uso de dados secundários, ressaltamos a importância da abordagem da IC numa perspectiva ecológica no presente estudo em relação ao dimensionamento do impacto da doença no SUS. A análise das internações como um dos desfechos da IC fornece dados relevantes para adequação das estratégias de controle ambulatorial da doença.

## Conclusões

Nosso estudo constatou tendência decrescente das taxas de internação por IC e crescente das taxas de mortalidade durante a internação em todas as faixas etárias avaliadas, para ambos os sexos, no período de 22 anos (2000 a 2021).

Os dados refletem a tendência temporal das internações por IC no Brasil durante o período analisado, permitindo que especialistas, gestores e epidemiologistas em saúde, com base nesse cenário, desenvolvam estratégias para a abordagem da doença e seus fatores de risco nas próximas décadas.
