# Supplementary material for: Tendência Temporal das Internações Hospitalares por Insuficiência Cardíaca no Brasil
Source: Arq Bras Cardiol. 2025 May 19;122(6):e20240505. [Article in Portuguese] doi: 10.36660/abc.20240505 (PMC12133065; doi:10.36660/abc.20240505)
Supplement: Supplementary file 1 [file 0066-782x-abc-122-06-e20240505-suppl01.pdf]

Tabela suplementar – Frequências absolutas de internações, taxas de internação e taxas de mortalidade na internação por insuficiência cardíaca em homens e mulheres, por faixas etárias, entre 2000 e 2021 no Brasil.

**Anos**	**Faixas etárias em anos**									
			**Homens**					**Mulheres**		
	**40 a 49**	**50 a 59**	**60 a 69**	**70 a 79**	**≥ 80**	**40 a 49**	**50 a 59**	**60 a 69**	**70 a 79**	**≥ 80**
**2000**										
N	18.355	34.072	50.805	51.530	29.953	17.511	31.397	47.516	53.330	37.354
TxI	19,3	55,9	131,7	238,8	479,1	17,4	47,5	106,6	191,6	421,9
TxM	5,2	5,3	5,9	7,0	9,2	4,0	4,8	5,7	7,0	10,3
**2001**										
N	17.398	33.024	48.226	50.835	30.151	17.083	29.982	45.271	52.328	37.313
TxI	17,8	52,1	121,8	228,9	460,2	16,4	43,6	98,8	181,9	392,3
TxM	5,5	5,0	5,8	7,1	9,2	4,1	4,9	5,5	6,8	10,4
**2002**										
N	16.506	31.943	46.520	49.513	30.165	15.497	28.377	43.283	51.393	37.035
TxI	16,3	48,4	114,3	219,1	425,1	14,4	39,5	91,9	175,7	350,6
TxM	6,2	5,6	6,3	7,4	9,1	4,2	4,6	6,0	7,5	10,7
**2003**										
N	16.007	30.652	45.058	47.889	29.228	14.581	26.932	40.488	48.861	36.003
TxI	15,4	44,7	107,7	208,8	380,3	13,1	36,0	83,6	164,8	309,6
TxM	5,6	5,6	6,4	7,8	9,7	4,3	5,0	6,2	7,5	11,3
**2004**										
N	14.977	29.656	43.843	46.028	28.292	13.279	24.429	38.137	46.764	35.108
TxI	14,0	41,5	101,8	197,3	342,3	11,6	31,3	76,5	155,2	277,6
TxM	6,0	5,8	6,7	7,9	10,4	4,8	5,4	6,4	8,0	11,8
**2005**										
N	14.323	28.131	40.664	42.596	26.569	11.826	22.896	34.950	43.387	33.130
TxI	13,0	37,9	91,5	178,6	302,2	10,1	28,1	68,0	141,1	244,8
TxM	5,9	5,7	6,4	8,1	10,7	4,4	5,3	6,6	8,0	11,9
**2006**										
N	13.432	26.578	38.056	40.466	26.491	10.793	21.466	32.287	40.864	32.750
TxI	11,9	34,5	82,8	165,3	285,3	9,0	25,4	60,8	129,5	228,6
TxM	5,9	6,0	6,9	8,3	11,0	4,7	5,6	6,9	8,4	12,5
**2007**										
N	13.189	26.321	37.356	39.193	25.842	10.930	20.801	31.412	40.599	32.844
TxI	11,5	33,0	78,5	155,2	264,8	8,9	23,6	57,1	124,7	218,3
TxM	5,8	5,9	7,0	8,4	11,1	5,2	5,7	6,7	8,4	12,4
**2008**										
N	11.596	24.390	34.250	35.830	24.124	9.455	19.126	28.657	36.411	30.070
TxI	9,9	29,5	69,4	137,1	234,5	7,5	20,9	50,3	108,1	188,5
TxM	6,3	6,4	7,0	8,7	11,3	5,7	5,8	6,8	8,6	12,6
**2009**										
N	11.655	24.799	34.058	36.134	24.588	9.571	19.331	28.871	36.805	31.051
TxI	9,7	29,0	66,5	133,6	225,7	7,5	20,4	48,7	105,7	181,4
TxM	6,2	6,5	7,5	9,0	12,0	5,4	6,0	7,1	8,7	13,4
**2010**										
N	11.207	24.263	32.959	35.338	24.676	8.915	18.742	27.989	35.708	31.197
TxI	9,2	27,4	61,8	126,4	214,9	6,9	19,1	45,4	99,3	170,8
TxM	6,4	6,6	7,8	9,6	12,5	5,9	6,2	7,9	9,3	13,7
**2011**										
N	10.926	24.193	32.954	34.560	24.466	8.906	18.473	27.579	35.384	31.104
TxI	8,8	26,5	59,5	120,1	205,9	6,8	18,2	43,0	95,6	163,8
TxM	6,7	6,5	8,1	9,9	13,4	5,6	6,7	8,0	9,6	14,6
**2012**										
N	10.026	22.452	31.262	32.412	22.861	7.881	17.315	25.630	32.733	29.706
TxI	8,0	23,9	54,3	109,5	185,8	5,9	16,6	38,3	85,9	150,5
TxM	6,7	7,0	8,2	10,1	13,5	5,7	6,6	8,1	9,5	15,0
**2013**										

N	9.630	21.686	30.357	31.371	22.579	7.607	16.582	25.010	31.294	29.129
TxI	7,6	22,4	50,8	102,8	177,2	5,6	15,4	35,9	79,7	142,0
TxM	7,0	7,0	8,3	10,3	13,8	5,9	7,0	8,1	10,1	14,8
**2014**										
N	8.965	20.089	29.416	29.215	21.164	7.081	15.451	24.140	29.956	28.095
TxI	7,0	20,2	47,4	92,8	160,1	5,2	14,0	33,3	73,9	131,6
TxM	6,7	6,9	8,4	10,2	14,6	5,5	6,6	8,3	10,2	15,4
**2015**										
N	8.377	19.718	29.409	29.270	20.434	6.707	14.915	23.762	28.885	27.690
TxI	6,5	19,3	45,6	89,9	148,9	4,8	13,2	31,5	68,9	124,6
TxM	7,3	7,1	8,7	10,9	15,1	6,0	7,7	8,8	10,7	16,5
**2016**										
N	8.204	19.380	29.340	28.680	20.299	6.376	14.476	23.377	28.320	26.873
TxI	6,2	18,5	43,9	85,1	142,1	4,5	12,5	29,8	65,2	115,9
TxM	8,2	7,6	9,1	11,4	16,3	6,6	7,7	9,4	11,5	17,1
**2017**										
N	7.743	18.259	28.047	27.925	20.442	6.006	13.820	22.878	27.810	27.276
TxI	5,8	17,1	40,5	79,8	137,1	4,2	11,7	28,1	61,7	112,6
TxM	7,2	7,6	8,9	11,4	16,1	6,1	7,3	9,1	11,2	17,0
**2018**										
N	7.622	17.328	27.094	27.670	19.600	5.683	12.827	21.957	26.801	26.240
TxI	5,6	15,8	37,7	76,1	125,8	3,9	10,6	26,0	57,3	103,6
TxM	7,0	7,9	9,2	11,7	16,2	6,6	8,1	9,4	11,3	17,2
**2019**										
N	7.473	17.371	27.411	27.446	19.602	5.732	12.704	21.640	26.214	26.395
TxI	5,4	15,6	36,9	72,6	120,7	3,9	10,3	24,8	53,8	99,9
TxM	7,5	8,1	9,4	12,0	16,8	7,2	8,1	9,6	11,9	17,4
**2020**										
N	6.918	15.440	23.882	22.973	16.022	5.034	10.841	18.365	21.855	21.807
TxI	4,9	13,6	31,1	58,3	94,9	3,3	8,7	20,3	43,0	79,2
TxM	7,5	8,2	10,0	13,0	17,5	7,8	8,7	10,6	12,7	18,6
**2021**										
N	6.554	14.903	22.787	21.973	15.557	4.659	9.914	17.652	21.302	21.489
TxI	4,5	12,9	28,7	53,5	88,7	3,0	7,8	18,9	40,1	75,1
TxM	8,5	9,2	11,8	14,1	20,0	7,8	9,6	11,6	14,5	20,3

N: número de internações por insuficiência cardíaca (IC) corrigidas por causas mal definidas; TxI: taxas de internação por IC por 10 mil habitantes; TxM: taxas de mortalidade na internação por IC, por 100 internações.

Elaborado pelos autores (2024); fonte: DATASUS[6].
