# Supplementary material for: Tendência Temporal das Internações Hospitalares por Insuficiência Cardíaca no Brasil
Source: Arq Bras Cardiol. 2025 May 19;122(6):e20240505. [Article in Portuguese] doi: 10.36660/abc.20240505 (PMC12133065; doi:10.36660/abc.20240505)
Supplement: Translation

# Translation: Temporal Trends of Hospital Admissions Due to Heart Failure in Brazil

**DOI:** 10.36660/abc.20240505i

**Authors:** José Marcos Girardi, Isadora Araújo Girardi, Ana Clara Silva Nascimento, Daniel Monteiro de Lauro Silva, Luisa Venture Gibaile Soares, Sarah Alessandrini Lauriano Dias, Sarah Quick Lourenço de Lima, Flávia Araújo Girardi

**Affiliations:** 1 Brazil Sociedade Brasileira de Cardiologia – Cardiologia, Juiz de Fora, MG – Brazil; 2 Brazil Faculdade de Ciências Médicas e da Saúde de Juiz de Fora, Juiz de Fora, MG – Brazil; 3 Argentina Universidad Maimónides - Facultad de Medicina, Buenos Aires – Argentina; 4 Brazil Universidade Federal de Juiz de Fora, Juiz de Fora, MG – Brazil

**Keywords:** Heart Failure, Hospitalization, Mortality

## Abstract

**Background:**

Heart failure is a global pandemic that causes a significant reduction in quality of life, with an impact on hospital expenses. It is important to know the temporal trends of hospitalizations and mortality in order to outline coping strategies.

**Objective:**

The objective of this study was to describe the temporal trends of hospitalizations due to heart failure and mortality during hospitalizations between 2000 and 2021 in Brazil.

**Methods:**

We conducted a study on temporal trends in the rates of hospitalization and mortality during hospitalization, using data from the Department of Information Technology of the Brazilian Unified Health System, by means of joinpoint regression. We calculated the annual percentage change of the rates with respective 95% confidence intervals and alpha significance level of 0.05.

**Results:**

We analyzed 2,851,437 hospitalizations in men and 2,749,424 hospitalizations in women between 2000 and 2021 in Brazil. Regarding hospitalization rates, there was an annual percentage decrease of 6.7% to 8.1% in men and a decrease of 7.5% to 8.3% in women. With respect to mortality rates, there was an annual percentage increase of 1.8% to 3.6% in men and an increase of 3.1% to 3.5% in women.

**Conclusion:**

We observed a decrease in the rates of hospitalization due to heart failure and an increase in the mortality rates in all age ranges assessed, in both sexes, between 2000 and 2021 in Brazil. These findings may reflect better disease control and hospitalization only for more severe cases, but we emphasize the need for continued management of risk factors for the disease.

## Introduction

Heart failure (HF) is defined as a clinical syndrome in which the heart is not able to pump blood to meet tissue metabolic needs or is able to do so only at high filling pressures.^1^The most common causes of HF include ischemic heart disease, myocardial infarction, systemic arterial hypertension, and valvular heart disease.^2^

HF is considered a global pandemic, having affected approximately 64 million people worldwide as of 2017.^3^It causes a significant reduction in quality of life, with a significant impact on hospital expenses and emergency care.^1^In the United States, although the absolute number of patients with HF has increased, partially as a result of the increased number of elderly people, the incidence of HF has decreased. There was a decrease in hospitalizations due to HF up until 2012 in the United States. However, an increase was observed between 2013 and 2017, with 1.2 million hospitalizations due to HF recorded among 924,000 patients diagnosed with HF in 2017, representing a 26.0% increase during the period.^2^

In the United States, approximately 115 million people have hypertension; 100 million have obesity; 26 million have diabetes, and 125 million have atherosclerotic cardiovascular disease (CVD). These are well-known and high population attributable risk factors for the development of HF. Therefore, a large proportion of the population can be categorized as being at risk for HF.^2^In Latin America, a distinct clinical profile is found, with potential risk factors resulting from low investment in health, inadequate access to health care, and insufficient follow-up in primary or tertiary services.^1^

In this context, in Brazil, annual data compiled and research on the epidemiology of CVDs have shown that they tend to be the leading cause of death, coronary artery disease being the number one cause, followed by stroke. However, in 2021, COVID-19 became the leading cause in both sexes. These epidemiological data, reported by Cardiovascular Statistics – Brazil, revealed that self-reported diagnoses of hypertension, hypercholesterolemia, and diabetes mellitus occurred in 26.3% (2021), 14.6% (2019), and 9.1% (2021) of adults, respectively. There was a decrease in the number of cases of unknown diabetes in Brazil, which may have occurred due to the higher screening rate and greater access to diagnosis in some segments of the population. In 2021, obesity was recorded in 22.4%, with an increasing trend from 2006 to 2021. The prevalence of smoking in this population segment showed a 0.7% decrease between 2019 and 2021 in both sexes. With respect to physical activity, the survey observed that, in spite of the growing knowledge about the cardiovascular benefits of regular practice and a trend towards reducing physical inactivity among Brazilians in recent years, almost half of the population did not reach the minimum recommended level of physical activity.^4^

In spite of advances in HF treatment, the syndrome remains a serious pathology, whose survival 5 years after diagnosis may reach only 35.0%.^1^In Brazil, poor adherence to basic therapy for HF is the main cause of rehospitalizations and the elevated in-hospital mortality rate.^5^

The objective of this study was to describe the temporal trends of hospital admissions due to HF, as well as mortality during hospitalization, between 2000 and 2021 in Brazil, analyzing their distribution by age ranges.

## Methods

### Study type and data sources

This was an ecological study of temporal trends in HF hospitalization rates and mortality rates during hospitalization between 2000 and 2021 in Brazil.

We used data from the Department of Information Technology of the Brazilian Unified Health System (DATASUS, acronym in Portuguese), which directly assists the Brazilian Ministry of Health by providing support to information systems.^6^

### Study population and variables of interest

The study population included HF hospitalizations in patients of both sexes between 2000 and 2021 in Brazil. This period was chosen based on the availability of population-based data in DATASUS. Age ranges 40 years and older were selected, seeing that they accounted for more than 95.0% of HF hospitalizations in adults.

We used overall hospital morbidity data from the Brazilian Unified Health System (SUS, acronym in Portuguese), by place of residence, extracted from the DATASUS TABNET consultation tool.^7^The variables sex, age ranges (grouped in 10-year intervals) 40 years of age and older, number of hospitalizations, and mortality rates during hospitalization were selected for each year separately. In the tenth edition of the International Classification of Diseases (ICD-10) morbidity list,^8^the term “heart failure” was selected. Data on the resident population were extracted from the demographic and economic variables section of the TABNET tool, stratified by sex, age range, and years.

The outcome variable of mortality rate during hospitalization was extracted directly from the TABNET tool, where it was already calculated per every 100 hospitalizations. This is the ratio between the number of deaths and the number of approved hospital admission authorizations, calculated as hospitalizations, during the period, multiplied by 100.

The outcome variable of hospitalization rate was calculated as follows:


$$\frac{\text{Number of HF hospitalizations by age range, sex, and year}\;\times 10.000}{\text{Number of residents in the same age range, sex, and year}}$$

For the purpose of correcting the number of hospitalizations due to ill-defined causes (IDC), the adjustment proposed by Mathers et al.^9^was performed, based on which the percentage of hospitalizations due to ill-defined causes (PHIDC) was calculated, as follows:


$$\frac{Total\;hospitalizations\;–\;hospitalizations\;due\;to\;external\;causes}{(Total\;hospitalizations\;–\;hospitalizations\;due\;to\;external\;causes)\;–\;hospitalizations\;due\;to\;IDC}$$

Subsequently, a correction factor (CF) was calculated by age range and year, which was multiplied by the number of corrected hospitalizations:

CF of hospitalizations due to IDC:


$$\mathrm{CF}=1+\frac{\left(\mathrm{PHIDC}-1\right)}{2}$$

These corrections were performed for each sex, age range, and year. IDC are listed in ICD-10 in Chapter XVIII, between codes R00 and R99. Hospitalizations due to external causes are listed directly in the hospital morbidity section of the TABNET tool.

### Data analysis

We calculated the annual percentage change (APC) in HF hospitalization rates and in-hospital mortality rates by means of joinpoint regression modeling, using the calendar year as an independent variable, according to age ranges. This method makes it possible to identify trend change points over time and, based on the definition of the best model, APCs are calculated for each segment. Accordingly, the trend can be described and quantified, assessing whether it is statistically significant.

As a measure that summarizes the trend over the entire period, the average annual percentage change (AAPC) was used. For APC and AAPC, 95% confidence intervals were estimated, and an alpha significance level of 0.05 was applied. We selected the options of homoscedasticity, logarithmic transformation of the dependent variable, and a model for adjusting correlated errors based on the data. For trend analyses, 1-year periods were used, and the results were presented in the form of joinpoint regression graphs and tables.

Excel (Microsoft Office Home and Student 2019) was used for data entry, and Joinpoint Regression Software, version 4.9.0.0^10^was used for joinpoint regression analysis.

## Results

In the year 2000 in Brazil, 184,715 HF hospitalizations were registered among men and 187,108 among women, and the age range from 60 to 79 years represented, respectively, 55.4% and 53.9% of the absolute numbers of hospitalizations. However, hospitalization rates were higher in patients age 80 years or older, 479.1 in men and 421.9 in women. Mortality rates during hospitalization were also higher in this age range, 9.2 among men and 10.3 among women. In 2021, 81,744 HF hospitalizations were registered among men and 75,016 among women. The age range from 60 to 79 years represented 54.7% of hospitalizations in men, and the age range 70 years or older represented 57.0% of hospitalizations in women. Patients age 80 years or older had the highest hospitalization rates, 88.7 in men and 75.1 in women, as well as the highest mortality rates, 20.0 in men and 20.3 in women (Supplementary Table).

HF hospitalization rates showed a statistically significant decrease in all age ranges assessed in both sexes between 2000 and 2021. The averages for annual percentage decrease in HF hospitalization rates in men ranged from 6.7% for the age range between 40 and 49 years to 8.1% for the age range 80 years or older. Among women, these averages showed a 7.5% decrease in the age range between 70 and 79 years and an 8.3% decrease in the age range between 50 and 59 years (Central Figure and Table 1 ).

**Table 1:** – Average annual percentage change in the rates of hospitalization and mortality during hospitalization for heart failure in men and women, by age range, between 2000 and 2021 in Brazil

Age range/rates	Men			Women		
	AAPC	(95% CI)	Trend	AAPC	(95% CI)	Trend
40 to 49 years						
Hospitalization	–6.7%*	(–6.8; –6.6)	Decreasing	–8.0%*	(–8.2; –7.7)	Decreasing
Mortality	1.8%*	(1.5; 2.1)	Increasing	3.4%*	(3.1; 3.8)	Increasing
50 to 59 years						
Hospitalization	–6.9%*	(–7.0; –6.8)	Decreasing	–8.3%*	(–8.6; –8.1)	Decreasing
Mortality	2.7%*	(2.3; 2.8)	Increasing	3.4%*	(3.1; 3.7)	Increasing
60 to 69 years						
Hospitalization	–7.0%*	(–7.2; –6.9)	Decreasing	–8.1%*	(–8.4; –7.9)	Decreasing
Mortality	3.3%*	(2.9; 3.5)	Increasing	3.5%*	(3.0; 3.7)	Increasing
70 to 79 years						
Hospitalization	–7.0%*	(–7.2; –6.7)	Decreasing	–7.5%*	(–7.8; –7.3)	Decreasing
Mortality	3.4%*	(3.1; 3.5)	Increasing	3.5%*	(3.0; 3.7)	Increasing
80 years or older						
Hospitalization	–8.1%*	(–8.3; –7.8)	Decreasing	–8.2%*	(–8.4; –8.0)	Decreasing
Mortality	3.6%*	(3.4; 3.9)	Increasing	3.1%*	(2.8; 3.3)	Increasing

Hospitalization: rates of hospitalization per 10,000 inhabitants; mortality: rates of mortality during hospitalization per 100 hospitalizations. AAPC: average annual percentage change (2000 to 2021); CI: confidence interval. *Significantly different AAPC applying alpha significance level = 0.05. Prepared by the authors (2024).

HF hospitalization rates showed a decrease that varied according to the period and age range. During the first decade assessed, the most pronounced annual decreases were observed in the age ranges 80 years or older (8.9% between 2000 and 2007) and 60 to 69 years (8.7% between 2004 and 2008) for the male population. Among women, the most significant decreases were observed in the age ranges from 40 to 49 years (10.6% between 2000 and 2006), 50 to 59 years (9.9% between 2000 and 2007), 60 to 69 years (9.0% between 2000 and 2008), and 80 years or older (10.5% between 2000 and 2005). During the second decade, in general, the decreases in HF hospitalization rates were smaller, except for in men from 50 to 59 years (8.1% between 2011 and 2014). However, the most pronounced decreases in hospitalization rates were observed between 2019 and 2021, exceeding 11.0% per year from the age ranges 60 years and older for men and 12.0% from the age ranges 50 years and older for women ( Figures 1 and 2 ).

Mortality rates during HF hospitalization showed a statistically significant increase in all age ranges assessed in both sexes between 2000 and 2021. The average annual percentage increase in men ranged from 1.8% for the age range between 40 and 49 years to 3.6% for the age range 80 years or older. Among women, these means ranged from 3.1% for the age range 80 years or over to 3.5% for those between 60 and 79 years ( Central Figure and Table 1 ).

**Figure 1:** – Observed and estimated joinpoint regression values for hospitalization due to heart failure in men, by age ranges, between 2000 and 2021 in Brazil. Dots: observed values; solid lines: estimated values. Annual percentage change (APC) values with respective 95% confidence intervals: 40 to 49 years: –7.6%* (–8.0; –7.3); –5.3%* (–7.1; –4.7); –6.8%* (–8.0; –6.4); –4.1%* (–5.0; –3.4); –8.6%* (–10.0; –6.8). 50 to 59 years: –7.6%* (–7.9; –7.4); –4.2%* (–5.5; –3.7); –8.1%* (–8.7; –7.0); –5.7%* (–6.1; –4.5); –8.7%* (–10.4; –6.9). 60 to 69 years: –6.3%* (–6.9; –4.7); –8.7%* (–9.8; –7.9); –5.9%* (–6.1; –5.6); –11.2%* (–12.7; –8.9). 70 to 79 years: –4.0%* (–5.5; –0.8); –7.7%* (–9.2; –6.9); –6.1%* (–6.4; –5.3); –14.0%* (–16.1; –10.7). 80 years or older: –8.9%* (–10.0; –8.2); –6.4%* (–6.7; –5.8); –14.8%* (–17.3; –10.9). *Significantly different APC applying alpha significance level = 0.05. Prepared by the authors (2024).

**Figure 2:** – Observed and estimated joinpoint regression values for hospitalization due to heart failure in women, by age ranges, between 2000 and 2021 in Brazil. Dots: observed values; solid lines: estimated values. Annual percentage change (APC) values with respective 95% confidence intervals: 40 to 49 years: –10.6%* (–12.0; –9.6); –6.9%* (–7.1; –6.6). 50 to 59 years: –9.9%* (–10.8; –9.2); –6.7%* (–6.9; –6.2); –12.6%* (–15.0; –9.3). 60 to 69 years: –9.0%* (–9.9; –8.4); –6.6%* (–6.9; –5.8); –12.8%* (–15.6; –9.0). 70 to 79 years: –6.7%* (–6.9; –6.5); –14.6%* (–17.4; –10.2). 80 years or older: –10.5%* (–12.6; –8.7); –8.0%* (–11.0; –5.1); –6.1%* (–6.4; –5.0); –14.2%* (–16.7; –10.9). *Significantly different APC applying alpha significance level = 0.05. Prepared by the authors (2024).

For the male population, a more pronounced annual increase in hospitalization mortality rates was observed in the age ranges 60 to 69 years (11.8%) and 70 to 79 years (7.5%), between 2019 and 2021. Among women, the most pronounced annual increase was recorded in the age ranges 50 to 59 years (7.6%), 60 to 69 years (8.9%), and 70 to 79 years (9.3%) during the same period ( Figures 3 and 4 ).

**Figure 3:** – Observed and estimated joinpoint regression values for mortality during hospitalization for heart failure in men, by age ranges, between 2000 and 2021 in Brazil. Dots: observed values; solid lines: estimated values. Annual percentage change (APC) values with respective 95% confidence intervals: 40 to 49 years: 1.8%* (1.5; 2.1). 50 to 59 years: 2.3%* (1.2; 4.3); 5.9%* (2.3; 8.1). 60 to 69 years: 2.7%* (2.4; 6.1); 0.9% (–0.9; 2.7); 11.8%* (6.2; 15.3). 70 to 79 years: 2.9%* (2.6; 3.1); 7.5%* (3.5; 9.2). 80 years or older: 3.6%* (3.4; 3.9). *Significantly different APC applying alpha significance level = 0.05. Prepared by the authors (2024).

**Figure 4:** – Observed and estimated joinpoint regression values for mortality during hospitalization for heart failure in women, by age ranges, between 2000 and 2021 in Brazil. Dots: observed values; solid lines: estimated values. Annual percentage change (APC) values with respective 95% confidence intervals: 40 to 49 years: 4.0%* (3.4; 5.3); –0.3% (–3.1; 2.1); 4.7%* (3.5; 8.4). 50 to 59 years: 3.0%* (2.4; 3.2); 7.6%* (3.2; 10.3). 60 to 69 years: 2.9%* (2.3; 3.2); 8.9%* (3.3; 11.7). 70 to 79 years: 2.9%* (2.5; 3.1); 9.3%* (3.5; 11.4). 80 years or older: 3.1%* (2.8; 3.3). *Significantly different APC applying alpha significance level = 0.05. Prepared by the authors (2024).

## Discussion

Our study found a decreasing trend in HF hospitalization rates between 2000 and 2021 in all age ranges assessed, for both men (6.7% to 8.1%) and women (7.5% to 8.3%). Mortality rates during hospitalization showed an increasing trend during the same period in all age ranges assessed, for both men (1.8% to 3.6%) and women (3.1% to 3.5%).

HF is a growing economic and public health burden worldwide, mainly due to population aging. In Brazil, HF and stroke were the most frequent causes of hospitalizations due to circulatory system diseases in both sexes in 2021, each accounting for approximately 17.0% of the total. Among hospitalizations for all causes, 2.6% of cases were attributed to HF in the same year.^7^

According to DATASUS, 3,454,570 HF hospitalizations were recorded from 2008 to 2021, more than a third of the total clinical admissions related to cardiovascular conditions. However, there was a decrease in the number of clinical admissions for HF, which went from 298,474 (157 per 100,000) in 2008 to 181,441 (85 per 100,000) in 2021. This decrease was uniform over the years, and it was not correlated with a reduction in healthcare costs. The decreased number of admissions and increased expenses represented higher costs per admission during the observed period (from 912 Brazilian reals in 2008 to 1,787 Brazilian reals in 2021).^4^

The most common precipitating factors for hospitalization of patients with acute decompensation of HF include acute coronary syndrome, atrial fibrillation and other arrhythmias, superimposed cardiac disease (such as endocarditis), acute infections (such as pneumonia or urinary tract infection), non-adherence to diet or medication, anemia, hyper- or hypothyroidism, medications that increase sodium retention (such as nonsteroidal anti-inflammatory drugs), and medications with negative inotropic effects (for example, verapamil).^2^

We observed a decrease in HF hospitalization rates, which could be interpreted as a result of successful health policies in Brazil. It is worth underscoring, however, that the presence of classic risk factors (systemic arterial hypertension, dyslipidemia, obesity, sedentary lifestyle, smoking, diabetes mellitus, and family history of CVD) increases the likelihood of CVD, especially of ischemic etiology, emphasizing the importance of primary and secondary prevention. In a Brazilian study conducted between 2011 and 2012, the ischemic etiology of HF predominated in patients from the South (33.6%), Southeast (32.6%), and Northeast (31.9%) Regions. In the North Region, hypertensive etiology predominated (37.2%), and in the Center-West Region, Chagas disease (42.4%) predominated.^5^Ischemic heart disease remains the leading cause of death in both sexes in Brazil, despite the decline in the prevalence and incidence of the disease over the past 20 years.^11^In 2021 in Brazil, the highest mortality rates due to ischemic heart disease were attributable to systemic arterial hypertension, around 37.6 per 100,000 inhabitants, followed by rates attributable to high serum levels of low-density lipoprotein cholesterol, 26.7 per 100,000 inhabitants. However, these rates remained stable between 1990 and 2021. Regarding the rates of mortality due to hypertensive heart disease, the main associated risk factors were systemic arterial hypertension (13.3 per 100,000 inhabitants) and elevated body mass index (7.5 per 100,000 inhabitants), however, with a 17.0% and 36.0% increase in mortality, respectively, between 1990 and 2021.^12^Regarding Chagas disease, socioeconomic and environmental factors, housing conditions, and sanitation are still elements that impact the transmission of the disease in Brazil.^13^Thus, although some risk factors have shown to be stabilized, others have increased, with an impact on mortality due to heart diseases that lead to the syndrome of HF.

Another important aspect to consider is the advent of drugs capable of modifying the clinical course of the syndrome, reducing the risk of hospitalization. Until 1987, the treatment of HF included a low-sodium diet, diuretics for manifestations of congestion, digoxin, and rest. However, none of these recommendations had an epidemiological or clinical basis; rather, they were based merely on the pathophysiological knowledge at that time, the pharmacological effects of the medications, and common sense.^14^In 1987, a new era in HF treatment began, with the publication of the first randomized clinical trial using enalapril. This new drug was able to inhibit the renin-angiotensin-aldosterone system, a hormonal axis involved in the cause, maintenance, and increased risk of HF.^15^In 1996, betablockers were identified as the second class of drugs capable of changing the natural history of the disease.^16^In 1999, low-dose spironolactone, exploring its hormonal effect more than its diuretic effect, was the next drug to demonstrate a definitively proven benefit for HF prognosis.^17^It was followed by angiotensin receptor-neprilysin inhibitors in 2014^18^and sodium-glucose cotransporter 2 inhibitors in 2019,^19 , 20^which were considered innovative drugs in HF treatment because they demonstrated significant reductions in the risk of hospitalizations of approximately 40.0% to 45.0% and 30.0%, respectively.^14^Various medications are currently available through the SUS for HF treatment, including those with an impact on survival, such as angiotensin-converting enzyme inhibitors, betablockers, and aldosterone antagonists.^21^

In our study, more pronounced decreases in HF hospitalization rates were also observed between 2019 and 2021, which may have been due to the SARS-CoV-2 pandemic. Measures with the aim of reducing the transmission of COVID-19 affected the organization of health services, with reduced in-person care and recommendations that the population seek services only in cases of extreme necessity.^22 , 23^

In our study, there was an increase in mortality rates during HF hospitalizations between 2000 and 2021 in Brazil, which could reflect the fact that only more severe cases were hospitalized or the high frequency of comorbidities, especially in elderly patients.^1^In Brazil, in-hospital mortality rates for HF were observed to be twice as high compared to records from the United States and Europe.^4^Deaths attributable to heart diseases have increased globally, in part due to increased recognition, diagnosis, and documentation of specific cardiomyopathies and cases of cardiotoxicity.

We also observed a more pronounced increase in the rates of mortality during HF hospitalization in the age ranges from 60 to 79 years between 2019 and 2021. In 2020, we found a decrease in the absolute number of deaths due to CVD in Brazil, albeit with an increase in hospital mortality in all regions of the country, which may be related to several factors. Changes in the health system during the pandemic, for example, redirecting teams to care for patients with COVID-19, resulted in a reduction in consultations and elective surgeries. Furthermore, the delay in seeking medical care and the harmful effects of SARS-CoV-2 infection on the cardiovascular system may have contributed to increased clinical decompensation of patients with heart disease.^22^

With the pandemic, researchers have obtained better information about infection and myocardial injury related to inflammation and myocarditis. With the increasing ability to detect myocardial injury using biochemical and imaging diagnostic methods, in addition to a growing awareness of patterns of cardiotoxicity and injury, including inflammation, the incidence of HF will likely continue to increase.^2^

In the context of controlling risk factors, early diagnosis, adherence to HF treatment, and primary health care by the SUS play a fundamental role due to the longitudinal nature of care.^15^Furthermore, the implementation of health policies, for example, encouraging healthy lifestyle habits, primary and secondary prevention measures, and access to treatment for cardiovascular events, is an essential factor for controlling CVD worldwide.

Regarding the limitations of this study, it is worth highlighting that the characterization of HF in the DATASUS database may be underestimated, given that some hospitalizations may have been due to the underlying disease that led to HF or other comorbidities and, therefore, may have been recorded under a different ICD-10 code. Moreover, the possibility of underreporting warrants attention, as diagnosis of HF may not adequately identify all cases, given that imaging and biochemical analyses are not available in all services and regions of Brazil.

In spite of the limitations inherent to the use of secondary data, we emphasize the importance of approaching HF from an ecological perspective in this study with respect to measuring the impact of the disease on the SUS. The analysis of hospitalizations as one of the outcomes of HF provides relevant data for adapting outpatient disease control strategies.

## Conclusions

Our study found a decreasing trend in HF hospitalization rates and an increasing trend in mortality rates during hospitalization in all age ranges assessed, for both sexes, over the 22-year period (2000 to 2021).

These data reflect the temporal trend of HF hospitalizations in Brazil during the period analyzed, allowing health specialists, managers, and epidemiologists, based on this scenario, to develop strategies to manage the disease and its risk factors in the coming decades.
